# Editorial: Advances in the diagnosis and treatment of pediatric hematological disorders

**DOI:** 10.3389/fped.2026.1986635

**Published:** 2026-09-25

**Authors:** Yongsheng Ruan, Sixi Liu, Anthony Pak-Yin Liu, Weili Sun, Xuedong Wu

**Affiliations:** 1 Department of Pediatrics, Nanfang Hospital, Southern Medical University, Guangzhou, China; 2 Department of Hematology and Oncology, Shenzhen Children’s Hospital, Shenzhen, China; 3 Hospital for Sick Children, University of Toronto, Toronto, ON, Canada; 4 Oncology, Johnson and Johnson Innovative Medicine, Los Angeles, CA, United States

**Keywords:** blastic plasmacytoid dendritic cell neoplasm (BPDCN), bone marrow failure, congenital dyserythropoietic anemia, hemophagocytic lymphohistiocytosis (HLH), hereditary spherocytosis, leukemia

Pediatric hematology has entered into a precision era. The traditional diagnostics relying on clinical morphology are rapidly being supplanted or augmented by high-throughput genomic sequencing, advanced machine learning models, and immunotherapeutic salvage therapies. This research topic, entitled “Advances in the Diagnosis and Treatment of Pediatric Hematological Disorders,” brings together 14 papers that collectively redefine clinical management paradigms. Spanning four major scenarios—molecular leukemogenesis, prognostic and complication modeling, bone marrow failure and erythrocyte pathologies, and diagnostic pitfalls in rare malignancies—this Research Topic offers clinical researchers a comprehensive state-of-the-art overview of pediatric hematology.

## Scenario 1: molecular mechanisms and genetic susceptibility in pediatric leukemias

The genetic abnormalities of pediatric acute lymphoblastic leukemia (ALL) and acute myeloid leukemia (AML) are highly heterogeneous, often involving specific germline predispositions or complex fusion translocations that drive lineage plasticity and chemotherapy resistance.

In a remarkable case report, Li et al. demonstrated a 6-month-old monozygotic twin pair diagnosed with concurrent KMT2A::AFF1-positive B-ALL. The occurrence of synchronous leukemia with identical gene fusions in infants provides a unique window into prenatal leukemogenesis, strongly supporting the “placental-transfer hypothesis” wherein a pre-leukemic clone originates *in utero*. Interestingly, instead of proceeding to allogeneic hematopoietic stem cell transplant (allo-HSCT), the twins achieved sustained molecular remission for over 8 years following sequential targeted CD19- and CD22-chimeric antigen receptor T-cell (CAR-T) therapy bridged with chemotherapy. This provides a potentially transplant-sparing curative approach for this high-risk infant cohort.

Similarly, exploring genetic identity in twins, Xu et al. identified a novel germline mutation, ETV6 c.744del (p.S248fs), in identical twins who eventually developed B-cell ALL. This represents the first international report of this variant, which segregates heterozygous carriers (the mother and maternal uncle) from wild-type family members.

Addressing therapeutic resistance in Philadelphia chromosome-positive (Ph+) ALL, Li et al. elucidate the tumor-suppressive role of the purinergic receptor P2RX1. Using TARGET database analysis, they reveal that high expression of P2RX1 is associated with worse survival. Mechanistically, establishing P2RX1 overexpression in SUP-B15 cells triggers a significant influx of intracellular calcium (Ca^2^⁺) and activates calmodulin-dependent protein kinase II (CaMKII). This activation downregulates the downstream PI3 K/AKT pathway, compromises mitochondrial membrane potential, reduces ATP production, and triggers upregulation of pro-apoptotic proteins. The calcium-mitochondria-apoptosis axis represents a novel target to overcome tyrosine kinase inhibitor (TKI) resistance in Ph + ALL.

Lineage plasticity remains a substantial diagnostic and therapeutic hurdle. Alonso-Carballo et al. report a t(12;19)(p13;p13.3) translocated leukemia causing a TCF3::ZNF384 fusion in an 11-year-old girl presenting with central nervous system leukemia and monocytic B-cell aberrant markers, initially diagnosed as AML. Since TCF3::ZNF384 fusion initiates transcriptional programs and promotes B/myeloid lineage plasticity, patients are prone to dynamic lineage shifts. The patient achieves long-term molecular remission under the NOPHO-AML-2012 protocol and is paired with highly sensitive flow cytometry-based minimal residual disease (MRD) monitoring.

## Scenario 2: prognostic models, complication prediction, and supportive care in pediatric leukemia

As survival rates for pediatric leukemia exceed 85%, managing treatment-related toxicities and early complications has become crucial to maintaining chemotherapy intensity.

Focusing on newly diagnosed pediatric acute promyelocytic leukemia (APL) treated with all-trans-retinoic acid (ATRA) and arsenic trioxide (ATO), Fu et al. apply latent growth mixture modeling (LGMM) to capture early dynamic white blood cell (WBC) count trajectories over the first 7 days of induction in a retrospective cohort of 93 patients. They identify four distinct trajectories. Multivariate analysis demonstrates that Class 1 (high-level increasing trajectory) and Class 4 (low-level increasing trajectory) are significantly associated with an elevated risk of developing differentiation syndrome. Moreover, Class 1 patients also require significantly higher transfusion volumes of red blood cells (RBC), platelets, and plasma.

To prevent asparaginase-associated pancreatitis (AAP), a severe toxicity of PEG-asparaginase, Ding et al. develop and validate a nomogram using a retrospective cohort of 352 ALL patients treated with the CCLG-ALL-2018 protocol. Ten percent of the cohort developed AAP. Six independent clinical and laboratory risk factors are integrated: history of AAP, lower albumin levels, elevated AST, elevated total bilirubin, hypocalcemia, and hyperglycemia. The nomogram achieved excellent predictive accuracy with an area under the curve (AUC) of 0.926 and robust internal validation (corrected AUC of 0.923), providing a practical clinical decision tool for preventing AAP.

For the early detection of sepsis in ALL patients during chemotherapy-induced neutropenia (CIN), Wu et al. evaluate RBC parameters. In a retrospective cohort of 264 children matched via propensity score matching (PSM) to control for baseline confounders (yielding 108 patients), they demonstrate that red cell distribution width (RDW) and pSOFA scores are independent risk factors for sepsis, while hemoglobin (Hb) is a protective factor. Early sepsis triggers inflammatory mediators that inhibit erythropoietin, impairing iron metabolism and increasing RDW heterogeneity. Their combined model (Hb + RDW) exhibits an AUC of 0.812, outperforming single markers and the pSOFA score.

Relapse remains the leading cause of treatment failure in pediatric ALL. Chen et al. analyze relapse predictors and long-term salvage outcomes in 436 pediatric patients treated with the CCCG-ALL-2015 protocol. Notably, thrombocytopenia, BCR::ABL1 positivity, and persistent MRD on day 19 and day 46 are identified as independent risk factors for relapse. Furthermore, allo-HSCT achieved significantly superior overall survival compared to those treated with chemotherapy or CAR-T cell therapy alone (82.6% vs. 38.1%) in relapsed patients.

## Scenario 3: bone marrow failure and rare erythrocyte pathologies

Bone marrow failure syndromes and rare congenital anemias present significant diagnostic challenges due to overlapping clinical phenotypes and morphological features.

Li et al. investigate the somatic mutational landscape of 224 pediatric acquired aplastic anemia (AA). Somatic variants in myeloid neoplasm-associated genes are detected in 20.5% of pediatric cases, primarily involving TET2 (19.6%), ASXL1 (10.9%), and MPL (10.9%), which are enriched in tumor-related metabolic and transcriptional regulatory pathways. In addition, mutation status does not impact immunosuppressive therapy efficacy, and no clonal evolution to MDS/AML was observed during follow-up.

Congenital dyserythropoietic anemia type IV (CDA IV) is a rare erythroid disorder caused by mutations in Krüppel-like factor 1 (*KLF1*), a master transcriptional regulator of erythropoiesis. Lu et al. present three pediatric cases harboring distinct mutations (c.525_526insCGGCGCC, c.1012C > T, c.1012C > A, and c.973G > A) who presented with severe hyperbilirubinemia, elevated HbF, and microcytic anemia from birth, requiring regular transfusions. Moreover, allo-HSCT using paternal or sibling donors successfully achieved stable donor chimerism, progressive immune reconstitution, and complete transfusion independence.

Diagnosing comorbid hereditary spherocytosis (HS) and idiopathic pulmonary hemosiderosis (IPH) is remarkably challenging. Li et al. present a 9-year-old girl with a 5-year history of refractory anemia, cough, and chest CT flocculent infiltration, who was diagnosed with IPH following positive BALF hemosiderin-laden macrophage screening. Although her hemoglobin initially recovered on oral prednisone, she subsequently developed indirect hyperbilirubinemia and jaundice. Subsequent peripheral smears revealed 28% spherocytes, and genetic testing identified compound heterozygous mutations in the ANK1, PKLR, and SPTB genes, confirming comorbid HS. The authors highlight the necessity of genetic testing in cases of unexplained chronic anemia.

## Scenario 4: diagnostic traps and novel paradigms in rare malignancies

The integration of novel computational models and precision therapies has redefined clinical workflows for rare malignancies.

Pediatric blastic plasmacytoid dendritic cell neoplasm (BPDCN) is a rare myeloid malignancy arising from plasmacytoid dendritic cells, frequently misdiagnosed as AML. Chen et al. analyze two BPDCN cases. While Case 1 achieved sustained remission using an ALL-type chemotherapy protocol followed by allo-HSCT, Case 2 was misdiagnosed as AML-M5. Subsequently, Case 2 relapsed eight months following diagnosis and further confirmed the diagnosis as BPDCN (HLA-DR, CD56, CD123, and CD304 +). Furthermore, Case 2 achieved a second complete remission utilizing CLL1-targeted CAR-T cell therapy as a bridge to successful allo-HSCT.

Further illustrating pediatric diagnostic challenges, He and Fu report a 12-year-old girl presenting with severe isolated thrombocytosis, without splenomegaly, anemia, or leukocytosis. Initially misdiagnosed with essential thrombocythemia, she failed conventional hydroxyurea and aspirin therapy. Despite a normal chromosome karyotype, qPCR detected high levels of the BCR::ABL1(P210) fusion transcript, confirming chronic myeloid leukemia (CML). Initiating imatinib therapy achieved a complete molecular response within 4 months.

Finally, pediatric hemophagocytic lymphohistiocytosis (HLH) is a life-threatening cytokine storm carrying a 20% to 25% early mortality rate. Wu et al. construct a 30-day mortality risk stratification model in 133 children with HLH. By integrating the Boruta feature selection algorithm with latent class analysis (LCA), they identify six key peripheral blood predictors: APTT, MCH, CNS involvement, cyclosporine treatment, CRP, and imaging hepatomegaly.

## Conclusion

The 14 articles compiled in this research topic collectively underscore that the future of pediatric hematology relies on diagnostic precision and personalized therapeutic algorithms. By integrating gene sequencing, machine learning-driven risk stratification, and targeted immunotherapies like CAR-T, pediatricians can systematically bypass conventional diagnostic blind spots, predict treatment complications, and improve overall survival.

